# Improvement of osteoblast adhesion, viability, and mineralization by restoring the cell cytoskeleton after bisphosphonate discontinuation *in vitro*

**DOI:** 10.1590/1678-7757-2024-0034

**Published:** 2024-08-12

**Authors:** Somying PATNTIRAPONG, Chunya CHAMPAKERDSAP, Pichaya MATHAVEECHOTIKUL, Apichaya VATANASILP

**Affiliations:** 1 Thammasat University Research Unit in Dental and Bone Substitute Biomaterials Faculty of Dentistry Thammasat University Pathumthani Thailand Thammasat University Research Unit in Dental and Bone Substitute Biomaterials, Faculty of Dentistry, Thammasat University, Pathumthani, Thailand.; 2 Thammasat University Faculty of Dentistry Pathumthani Thailand Thammasat University, Faculty of Dentistry, Pathumthani, Thailand.

**Keywords:** Discontinuation, Bisphosphonate, Osteoblast, Viability, Mineralization, Cell cytoskeleton

## Abstract

Bisphosphonates are prescribed to treat excessive bone resorption in patients with osteoporosis. However, its use is associated with potential adverse effects such as medication-related osteonecrosis of the jaw, prompting the introduction of the drug holiday concept in patients prior to dentoalveolar surgery. Furthermore, bisphosphonate discontinuation has been studied *in vivo*, in humans, and in animal models. However, it is not known whether this approach could affect bone cells *in vitro*. Therefore, the objective of this study was to investigate the potential effects of bisphosphonate discontinuation on pre-osteoblast and osteoblast activities *in vitro*.

**Methodology:**

Pre-osteoblasts (MC3T3) and osteoblasts were treated with bisphosphonate (alendronate) at concentrations of 1, 5, and 10 µM. Alendronate was then withdrawn at different time points. The negative control consisted of untreated cells (0 µM), while the positive control consisted of cells incubated with alendronate throughout the experiment. Cell viability, cell adhesion, cell cytoskeleton, mineralization, and gene expressions were investigated.

**Results:**

Pre-osteoblasts and osteoblasts showed a decrease in cell viability after treatment with 5-10 μM alendronate for 4 days or longer. Two days of alendronate discontinuation significantly increased cell viability compared with the positive control. However, these levels did not reach those of the negative control. Bone nodule formation was reduced by alendronate. Discontinuation of alendronate regained bone nodule formation. Longer periods of discontinuation were more effective in restoring nodule formation than shorter periods. Addition of alendronate resulted in an increase in the percentage of dead cells, which, in turn, decreased when alendronate was discontinued. Alendronate affected the cell cytoskeleton by disassembling actin stress fibers. Cell adhesion and cell morphological parameters were also affected by alendronate. Discontinuation of alendronate restored cell adhesion and these parameters. Overall, the highest improvement after alendronate discontinuation was seen at 10 µM. However, alendronate treatment and discontinuation did not affect osteoblast gene expression.

**Conclusion:**

Discontinuation of alendronate helps to reverse the negative effects of the drug on cell viability, cell adhesion, and mineralization by restoring the cell cytoskeleton. Our data suggest the benefits of drug holiday and/or intermittent strategies for alendronate administration at the cellular level.

## Introduction

Bisphosphonates (BPs) are anti-remodeling drugs prescribed for the prevention and treatment of postmenopausal osteoporosis.^1^ Alendronate (ALN) is an oral BP that is recommended as a first-line drug to patients because of its efficacy and cost-effectiveness.^2^ BP use has been associated with adverse effects, namely osteonecrosis of the jaw (ONJ) and atypical femoral fractures (AFF).^3-6^ In these conditions, bone resorption and bone formation are disrupted, especially at the affected sites. The rate of bone formation is reduced after long-term ALN administration in the case of AFF ^4^ and in healthy postmenopausal patients.^7,8^ As a result, prolonged suppression of bone turnover occurs.^4,6,8,9^ Osteoblasts are almost absent in the necrotic jawbone of patients with ONJ.^9^

Data from *in vitro* studies support the suppression of bone formation by ALN.^10,11^ ALN directly impairs bone nodule formation and osteoblast differentiation.^11,12^ It inhibits osteoblast cell growth and induces cell apoptosis and necrosis.^10-13^ In addition, pre-osteoblast adhesion is reduced by the addition of ALN.^13^ This osteoblast activity is disrupted, resulting in a net loss of bone formation.

The adverse effects of BPs have sparked interest in a “drug holiday” approach or temporary medication discontinuation after continuous long-term BP administration.^14,15^ This approach is used to prevent the development of ONJ or to accelerate healing in patients with established ONJ. Although evidence supporting or refuting this approach is unclear, the anti-remodeling effect of the drug has been shown to be reversed in patients who receive ALN for 2 years followed by 1 year of discontinuation.^7^ The “drug holiday” practice has shown an upward trend from 1.7% in 2010 to 14% in 2012 and has remained steady since then.^16^

While BP discontinuation has been studied in both humans and animals,^17-19^ to our knowledge, it has not been investigated *in vitro*. Since osteoblasts are some of the cells affected by ALN,^10,11,13^ ALN discontinuation may restore osteoblast viability and function and could be encouraging for managing/preventing undesirable conditions. In this study, we determined the effects of ALN discontinuation on pre-osteoblast and osteoblast activities at the cellular level. Cell viability, mineralization, cell adhesion, and gene expressions were investigated at various time points after ALN discontinuation. The duration of ALN discontinuation was also taken into account in some experiments.

## Methodology

### Cell culture

MC3T3 cells (CRL-2593), murine pre-osteoblasts, were obtained from the American Type Culture Collection (ATCC). These cells were cultured in alpha-minimum essential medium supplemented with 10% fetal bovine serum and 1% penicillin/streptomycin (standard culture medium; Gibco). For osteogenesis, MC3T3 cells were incubated in osteogenic medium (OM; standard culture medium containing 50 µg/ml ascorbic acid from BDH and 2 mM β-glycerophosphate from Sigma-Aldrich^®^ Lab & Production Materials).

Cells were plated at a density of 8x10^3^ cells/1 cm^2^. The negative control consisted of untreated cells (A0) cultured in standard media or OM. The positive control consisted of cells incubated with ALN (Sigma-Aldrich^®^ Lab & Production Materials) at 1, 5, and 10 µM (A1, A5, and A10, respectively) throughout the experiment. For the samples tested, cells were first treated with ALN, then the drug was withdrawn and replaced with fresh media. Treatment time and stop time (S) were indicated in each experiment.

### Cell viability assay

Cells were treated with three concentrations (A1, A5, and A10). Nine treatment groups were established with different treatment and drug discontinuation periods. All treated samples and their controls in each treatment group were terminated at the same endpoint. (Figure S1A). The media were discarded and 0.2% Thiazolyl Blue Tetrazolium Bromide powder (Sigma-Aldrich^®^ Lab & Production Materials) dissolved in fresh media was added and incubated at 37°C for 4 h. The reaction was stopped with dimethyl sulfoxide and glycine buffer. The color of the product was measured at 570 nm absorbance.

### Mineralized matrix formation assay

Osteoblasts were induced with OM for 20 days. Cells were treated with A1, A5, and A10. Figure S1B shows the time periods of ALN treatment and stoppage. The samples in which ALN treatment was stopped at 5, 10, and 15 days were abbreviated as S5d, S10d, and S15d, respectively. All treated samples and their controls were terminated at the same endpoint. Cells were fixed with ice-cold ethanol for 10 min and washed with phosphate-buffered saline (PBS). One percent alizarin red S (ARS; Sigma-Aldrich^®^ Lab & Production Materials) solution was added to stain the bone nodule formation for 2 min. Images of the entire well were captured using a stereo microscope (Nikon SMZ1000) with a 1x objective. The staining area was processed using ImageJ software (version 1.52a-Java 1.8.0).^12^ Cells were destained with 10% cetylpyridinium chloride (Sigma-Aldrich^®^ Lab & Production Materials), and the extracted stains were measured at 405 nm.

### Cell death, cell adhesion, and cell cytoskeleton assays

Pre-osteoblasts were treated with A5 and A10 for 4 days and discontinued for 3 days, and designated as A5S and A10S, respectively. One untreated group (A0) and two groups treated with ALN (A5 and A10) for 7 days served as negative and positive controls, respectively. Cells were trypsinized, stained with trypan blue, and counted for live and dead cells. Live cells from each sample were replated at the same density of 4,000 cells/well (48-well). Pre-osteoblasts were further incubated at 37°C for 5 h. The cells were rinsed with PBS to remove non-adherent cells. The remaining adherent cells were fixed with 4% paraformaldehyde (Sigma-Aldrich^®^ Lab & Production Materials) for 15 min. The cells were permeabilized with 0.1% Triton X-100 for 15 min and stained with TRITC-conjugated rhodamine phalloidin (Invitrogen) at 1:200 dilution for 1 hour and 4’,6-diamidine-2-phenylindole dihydrochloride (DAPI; Sigma-Aldrich^®^ Lab & Production Materials) at 1:1000 dilution for 5 min (Figure S1C). Images of the cells were captured with the same acquisition parameters at 200× magnification using a confocal microscope and NIS element software (Nikon Eclipse Ti; Nikon Instruments). Images were processed using ImageJ to assess the number of DAPI-positive nuclei. At least 245 cells per sample were analyzed for actin filament and morphological parameters.^10^

### Real-time polymerase chain reaction (RT-qPCR)

**Figure 1:**
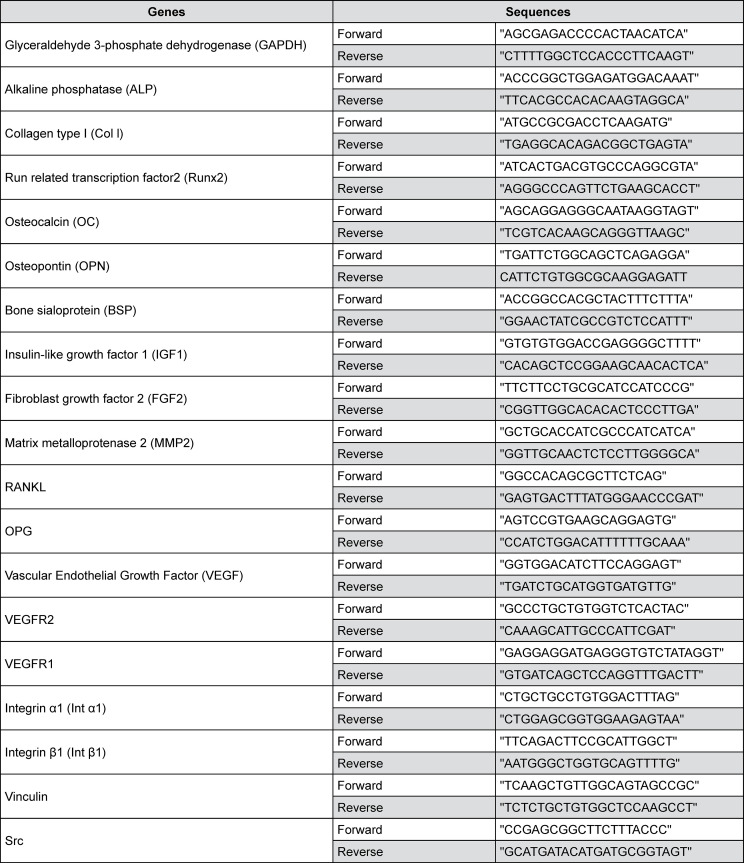
Primer sequences

Two treatment conditions were performed: 1) osteoblasts were treated with A5 for 1 day and discontinued for 1 day; and 2) osteoblasts were treated with A5 for 4 days and discontinued for 3 days. RNA was extracted using Total RNA Mini Kit (Geneaid Biotech Ltd). One microgram of mRNA from each sample was synthesized into cDNA using oligo dT (TAKARA BIO INC.). cDNA samples were amplified in a reaction mix containing KAPA SYBR^®^ FAST PCR Kit Master Mix (Thermo Fisher Scientific) and the forward and reverse primer pair sequences (Sigma-Aldrich^®^ Lab & Production Materials). Amplification was performed on QuantStudio™ 3 Real-Time PCR Systems (Thermo Fisher Scientific). Cycles were set at 50°C for 2 min of initial heating, 95°C for 1 min, 40 cycles of 95°C for 30 s, followed by 60°C for 30 s with 72°C extension for 30 s. Figure 1 shows the primer sequences. Quantitative PCR results were analyzed using the 2^-^_T_ method.^20^ The gene copy number was normalized with GAPDH. Data were expressed as fold change relative to A0.

### Statistical analysis

Normality was tested by the Shapiro-Wilk test using GraphPad Prism version 5 (GraphPad software). Data that were normally distributed were then analyzed by ANOVA, followed by Tukey’s test. Data that were not normally distributed were analyzed by Kruskal-Wallis, followed by Dunn’s multiple comparison test. Data were expressed as mean ± SD. Significance was set at *p<0.05, **p<0.01, and ***p<0.001 for comparisons with the sample in the same concentration group, and at #*p*<0.05, ##*p*<0.01, and ###*p*<0.001 for comparisons with A0.

## Results

### Effects of alendronate discontinuation on cell viability

**Figure 2:**
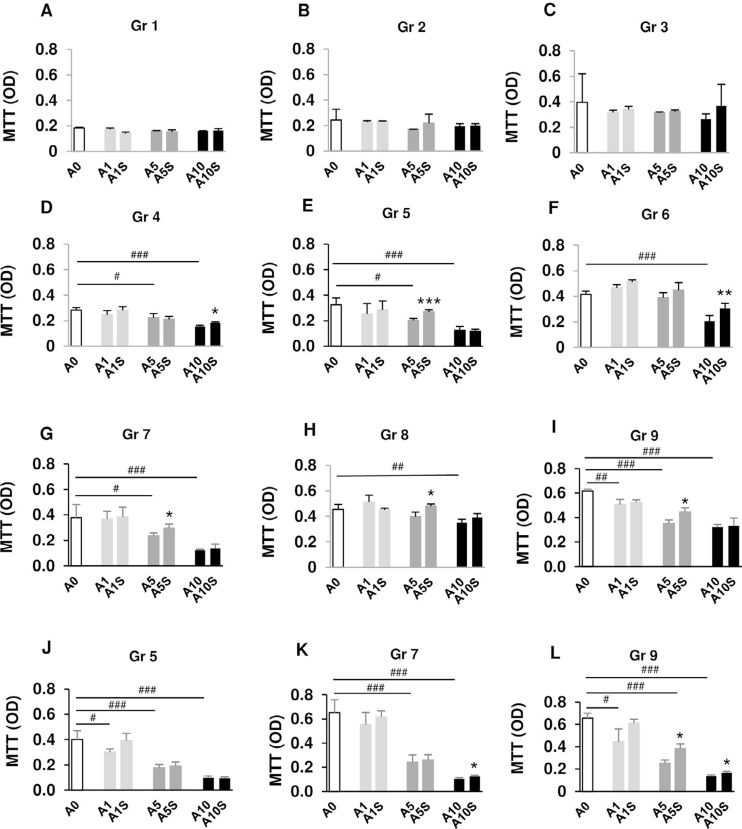
Cell viability of pre-osteoblasts and osteoblasts after ALN treatment and discontinuation at various time points. MC3T3 cells were incubated with 1, 5, and 10 µM ALN (A1, A5, and A10). Negative control was abbreviated as A0. The “S” was abbreviated for stoppage of ALN. N = 4

Pre-osteoblast viability of A0 gradually increased from 2 days to 7 days, indicating normal cell growth. Cells treated with ALN showed lower viability with the most effective dose at 10 µM (Figure S2). No significant alteration in viability was observed in Gr 1-3 after ALN treatment (Figure 2A-C). ALN treatment at 5-10 µM for more than 4 days reduced cell viability (Figure 2D-I). A decrease in cell viability was observed in Gr 9 at every ALN concentration (Figure 2I). A short period of ALN discontinuation did not improve cell viability (Figure 2A-C). ALN discontinuation significantly increased cell viability in the A5 group of Gr 5 and Gr 7-9 (Figure 2E and G-I) and in the A10 group of Gr 4 and 6 (Figure 2D and F) compared with the positive control.

Selected groups (Gr 5, 7, and 9) were later evaluated for osteoblast viability. ALN reduced osteoblast viability in a dose-dependent manner (Figure 2J-L), and no change in osteoblast viability was observed after 1 day of ALN discontinuation (Figure 2J). For Gr 7, A10 reduced viability by 83% compared with A0. ALN discontinuation for 2 days significantly increased viability in A10-treated osteoblasts of Gr 7 (Figure 2K). For Gr 9, A1-A10 inhibited osteoblast viability. Discontinuation of ALN for 3 days significantly increased cell viability in A5- and A10-treated osteoblasts of Gr 9, with the peak increase found at A5S by up to 52% of A5 (Figure 2L). Although cell viability levels of pre-osteoblasts and osteoblasts were increased after ALN discontinuation, these levels did not reach those of their respective untreated controls. These data suggest that discontinuation of ALN partially restored cell viability. A longer period of discontinuation resulted in a better outcome than a shorter period of discontinuation. However, the difference in results may be due to the duration of drug exposure.

### Restoration of bone nodule formation after alendronate discontinuation

**Figure 3:**
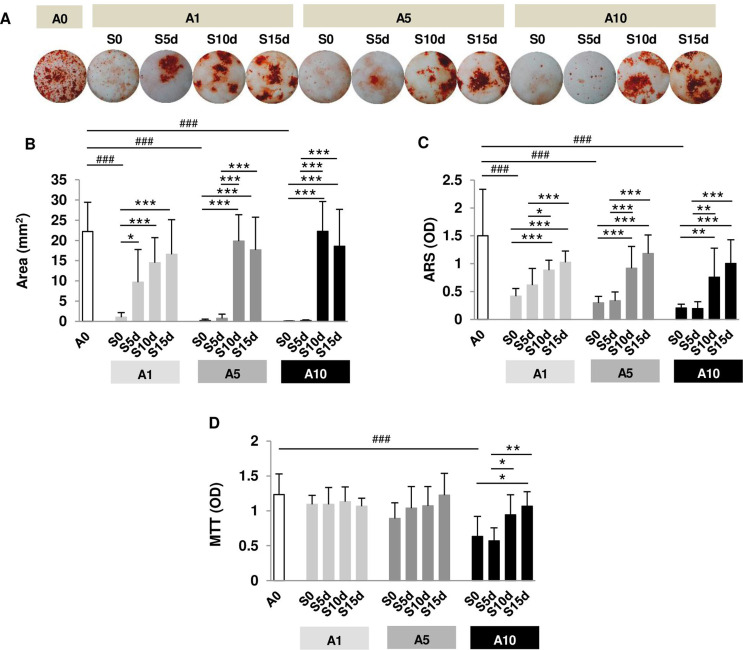
Examination of osteoblast nodule formation and viability. Osteoblasts were incubated with 1, 5, and 10 µM ALN (A1, A5, and A10). (A) Micrographs demonstrate nodule formation after treatment and cessation of ALN. (B) Quantification of alizarin red-positive area (mm2). (C) Quantification of alizarin red staining. (D) Osteoblast viability at 20 days. N = 8

The red staining area of bone nodule formation in A0 was distributed throughout the well. ALN addition reduced the red area, while ALN discontinuation restored the red area (Figure 3A). Nodule formation covered approximately 22 mm^2^ of the well surface (23% of the area) in A0 (Figure 3B). ALN treatment throughout the experiment (S0) significantly reduced the area of nodule formation. S5d increased nodule formation only in A1 (Figure 3B). Longer ALN discontinuation (S10d and S15d) substantially restored nodule formation in all concentrations tested (Figure 3B). ARS quantified by spectrophotometry showed the same trend as the mineralization area, except for S5d in A1 (Figure 3C). A longer discontinuation period significantly increased nodule formation compared with a shorter discontinuation period (Figure 3B and C).

Osteoblast viability was studied along with mineralization. A10 reduced osteoblast viability compared with A0. In A10, S15d significantly restored viability compared with S0 and S5d (Figure 3D). These data suggest that ALN at a high concentration inhibited osteoblast mineralization by partially affecting cell viability. Cell function was also affected by ALN (all concentrations). Furthermore, longer ALN discontinuation was more effective than short discontinuation in rescuing cell viability and mineralization.

### Improvement of cell adhesion, cell cytoskeleton, and cell morphology after alendronate withdrawal

**Figure 4:**
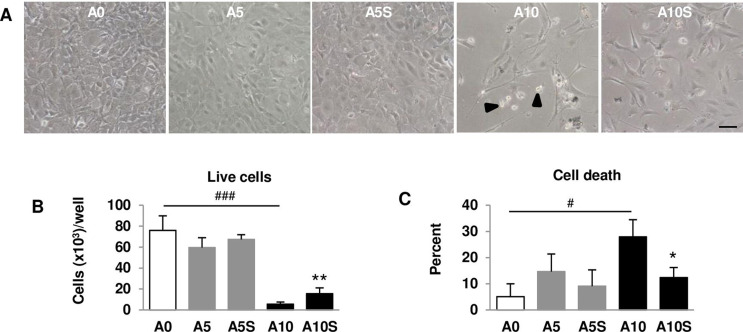
Evaluation of viable cells. (A) Micrographs of pre-osteoblast depicting cell morphology. (B) Number of live cells after 7-day cell culture. (C) Percent of dead cells after 7-day cell culture. Arrowhead indicated round cells. Scale bar = 100 µm. N = 4

While A0 cells had a normal shape, A10-treated cells showed altered shapes. Some A10-treated cells appeared spherical (indicated by arrowheads) and underwent disintegration. A10S cells had a polygonal and fibroblast-like appearance, with fewer round cells (Figure 4A). A0 cells were approximately 76,000 cells per well. A5 did not affect the number of live cells. Live A10 cell counts were significantly reduced compared with A0. A10S increased the cell number by 2.6-fold compared with A10 (Figure 4B). The percentage of cell death was low in A0. A10 increased cell death to 28%, while A10S significantly decreased cell death to 12% (Figure 4C).

**Figure 5:**
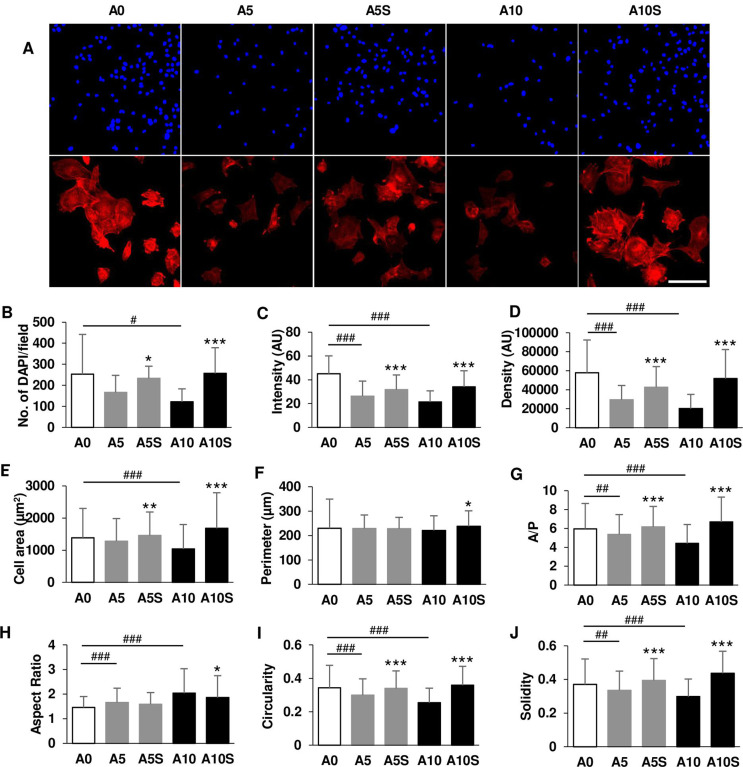
Investigation of cell adhesion after treatment and cessation of ALN. (A) Confocal images of nuclei and cell cytoskeleton. (B) Number of DAPI/field. (C) Stress fiber intensity. (D) Stress fiber density. (E) Cell area (µm2). (F) Cell perimeter. (G) Area/perimeter (A/P). (H) Aspect ratio. (I) Circularity. (J) Solidity. Scale bar = 100 µm.

After counting live and dead cells, live cells were processed as described in the Materials and Methods section to examine cell adhesion and cell cytoskeleton (Figure S1C). Nuclei are visualized in bright blue, while actin filaments are shown in red. The number of DAPI-positive nuclei represented the number of cells that adhered to the well plate after 5 h (Figure 5A; upper panel). Approximately 250 cells/field adhered to the plate in A0. A5 and A10 cell adherence was reduced by up to 50%. The number of attached cells increased significantly when ALN was removed (Figure 5B). While staining intensity and density of actin stress fiber were reduced in ALN-treated samples, stress fiber intensity and density recovered after ALN was discontinued (Figure 5A; lower panel, C and D).

Cells treated with ALN showed smaller cell spreading area, area/perimeter (A/P), circularity, and solidity compared with A0, while ALN discontinuation resulted in larger cell parameters (Figure 5E, G, I, and J). ALN did not affect cell perimeter, but A10S increased cell perimeter compared with A10 (Figure 5F). However, ALN increased aspect ratio (AR). Discontinuation of ALN at 10 µM reduced AR (Figure 5H). Additional statistical data are shown in Table S1. These data indicated that ALN lowered the rate of cell adhesion, interfered with actin filament organization, and affected cell morphology, but ALN withdrawal restored these parameters. These results may be due to the duration of ALN treatment.

### Effects of alendronate discontinuation on gene expression

**Figure 6:**
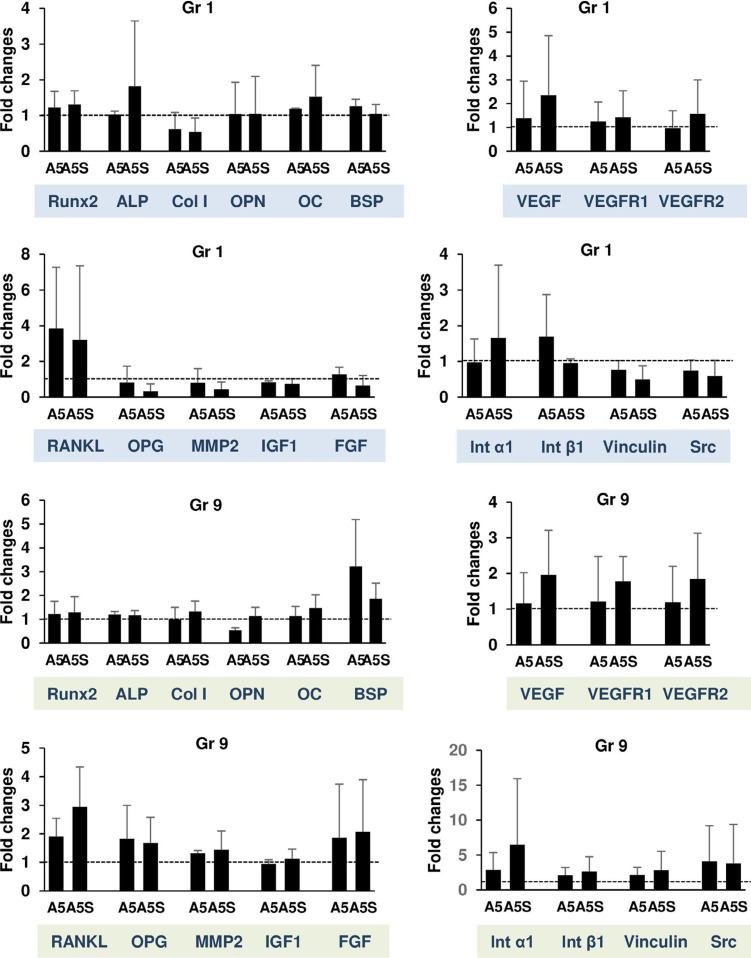
Gene expressions. Two conditions of treatment and cessation were investigated. Gr 1: Tx1d S1d, Gr 9: Tx4d S3d. Dashed lines are mRNA expressions of A0. N = 3

In this study, Gr 1 and 9 were selected for the examination of osteoblast mRNA expression because they were considered as short-term and long-term treatment and discontinuation. Specifically, the expressions of genes associated with osteogenesis, angiogenesis, mediators, and cell adhesion were examined. These genes included Runx2, ALP, Col I, OPN, OC, BSP, VEGF, VEGFR1, VEGFR2, RANKL, OPG, MMP2, IGF1, FGF, Int α1, Int β1, and Src. The mRNA expressions of A0 are shown as dashed lines. There was no difference in mRNA expressions of ALN-treated cells compared with the untreated and withdrawal groups (Figure 6).

## Discussion

After administration, BP circulates in the blood and rapidly accumulates in bones with high turnover, as documented in previous studies.^21,22^ In the oral cavity, BP is concentrated in the alveolar bone surrounding the roots of teeth, an area consistent with the occurrence of ONJ.^22^ Several cell types, including osteoblasts, inhabit the bone, and it is likely that osteoblasts are exposed to BP and capable of internalizing it.^12^ Internalization of BP disrupts cell viability and function,^10,12,23^ decreases bone formation, and prolongs the healing process; possibly leading to the development of ONJ.^24-26^

A subset of patients undergoing continuous oral bisphosphonate (BP) therapy may develop ONJ following tooth extraction^27^ and AFF.^4^ Rats obtaining continuous BP treatment also demonstrate ONJ with impaired bone formation and unhealed epithelium.^26^ Decreased bone formation is also observed in the affected femur obtained from AFF case.^4^ Hence, BP discontinuation is intended to prevent the risk of undesirable conditions such as ONJ in patient receiving long-term BP.^14,15^ Since osteoblasts are bone cells affected by BP,^10,13^ the application of the discontinuation approach was expected to cease and/or reverse the unwanted effect of BP on osteoblasts. While animal models have shown that osteoblast and osteoclast functions are restored after BP cessation,^18,28^ the information of discontinuation of ALN on osteoblast activities *in vitro* is still missing. This study showed the first evidence of the ALN discontinuation effects on osteoblasts in terms of cell viability, cell adhesion, cell cytoskeleton, mineralization, and gene expressions *in vitro*.

ALN regulates various aspects of osteoblast behavior, including viability, morphology, differentiation, and mineralization.^10-13,29^ This regulatory effect is attributed to ALN ability to mediate the organization of the actin cytoskeleton through RhoA. This is achieved by inhibiting farnesyl pyrophosphate synthase within the mevalonate pathway.^10,29^ The cell cytoskeleton organization is an early event, preceding several cellular activities.^10,30^ ALN destabilizes the actin filament, leading to reduction of viable cells and induction of cell death.^10,30^ Additionally, ALN can alter cell morphology by influencing cell cytoskeleton,^29^ a process recognized to indicate various stages of cell activities, such as viability, proliferation, differentiation, and migration.^10,31,32^ The disruption of actin cytoskeleton decreases osteoblast differentiation, alkaline phosphatase-positive matrix vesicles and bone mineralization.^30,33^ Consistent with previous studies,^10,13,29^ our results demonstrated that the addition of ALN affected actin filament, leading to cell morphological change, reduced cell adhesion, cell viability and mineralization (Graphical abstract). Since osteoblasts are present in the healing area of the socket from 7 days post-tooth extraction,^34^ interference with osteoblast activities during this critical period could potentially delay phases of bone healing.

Long-term administration of ALN has been observed to decrease the rate of bone formation in both humans and mice. However, withdrawal of ALN has been shown to restore the rate of bone formation and increase both osteoblast surface and mineralizing surface.^7,18^ These studies indicate that the reduction of bone formation can be reversed by cessation of BP. Removal of an inciting factor, as in ALN discontinuation, enables pre-osteoblasts to recover actin filament organization. In addition, the rate of cell adhesion was improved. ALN triggers the change in stress fiber density and adhesion molecule.^29^ Thus, some cells treated with ALN exhibited a reduced actin filament intensity and density, cell spreading area, as well as other cell parameters, including A/P, circularity, and solidity. Removal of ALN reversed these effects, cells demonstrated higher fiber density and extended well. Stabilization of actin cytoskeleton is found to enhance cell survival, osteoblast differentiation, and mineralization.^10,30^ Thus, the removal of ALN resulted in improved cell viability, adhesion, and function of osteoblasts (Graphical abstract).Discontinuing BP or adopting intermittent administration strategies may prove advantageous to mitigate the adverse effects of BP on osteoblasts.

Genes characterizing osteoblast markers and function such as ALP, Col I, OPN, OC, Runx2, IGF1, FGF2, RANKL, OPG, MMP2, and VEGF are upregulated under OM.^35^ These genes together with adhesion genes including Int α1, Int β1, vinculin, and Src did not alter after ALN treatment. As shown in previous reports, osteoblast marker genes have not been changed under A10 treatment.^35^ Discontinuation of ALN did not change these gene expressions; therefore, it was likely that the observed improvement in the net bone formation, after the cessation of ALN, was achieved through the restoration of cell viability, cell adhesion, and cell function, rather than alterations in gene expressions.

Duration of ALN treatment and discontinuation may play a role in the bone forming process. Longer treatments with ALN decreased cell survival to a higher extent than shorter periods. Treatment with ALN for 3 weeks decreased bone nodule formation more than treatment for 1-2 weeks.^36^ Generally, discontinuation of ALN for 2 days or longer partially rescued cell viability of pre-osteoblasts and osteoblasts. Discontinuation of ALN at longer periods regained cell viability and mineralization more than shorter periods. If possible, extending the period of ALN discontinuation is suggested to obtain a better outcome. It could be inferred that the higher regaining of nodule formation during the longer cessation period might be attributed to the examined cells being previously exposed to a shorter ALN treatment duration. Duration of BP treatment and cessation is considered crucial in wound healing process in patients with a long-term BP therapy; indeed, patients receiving oral BP for more than 5 years exhibit delayed healing of extraction sockets compared to those treated for a shorter duration.^25^ Prolonged drug holidays have been shown to enhance bone and epithelial healing, subsequently reducing incidence and severity of ONJ conditions.^26^

The concept of a drug holiday has been implemented by professionals but it lacks sufficient evidence to either support or refute this approach in patients. Therefore, more research is warranted.^27^ From a cellular perspective, drug holidays and intermittent administration approaches could mitigate the adverse effects of BP on bone cells, at least *in vitro*. BP discontinuation may contribute to improve osteoblast viability and function through the recovery of cell cytoskeleton. Longer periods of cessation might be necessary for full recovery of osteoblasts, thereby reducing risk of long-term adverse effects of BP, such as those previously mentioned in this study.

We acknowledge the limitations of this study and questions left unanswered. Firstly, it was an *in vitro* experiment, and the data may not directly reflect what really happens *in vivo*. Secondly, the *in vivo* system is more complex than the *in vitro*, and the release of BP from bone tissue into the bone microenvironment over several years, even after BP treatment cessation,^37^ was not considered in this *in vitro* study. Finally, the design of discontinuation duration was based on regular experimental assays and controls in each experiment, not on the duration of drug holiday in patients. The results could be attributed to the duration of drug treatment.

## Conclusion

This finding showed that discontinuation of ALN reversed the inhibitory effects of ALN on osteoblast viability, bone mineralization, cell cytoskeleton and adhesion. Applying the drug holiday concept has shown potential to facilitate recovery of ALN target cells, pre-osteoblasts and osteoblasts.
